# Oral supplementation of sodium butyrate prevents lipid metabolism disorders and intestinal injury by modulating immunity, intestinal barrier functions, and gut microbiota in a corticosterone-induced chronic stress model in mice

**DOI:** 10.1007/s44154-026-00289-2

**Published:** 2026-03-30

**Authors:** Fei Li, Lijun Wang, Ji Cheng, Shifeng Pan, Yao Lu

**Affiliations:** 1 College of Veterinary Medicine, Yangzhou University, Yangzhou, Jiangsu 225009 People’s Republic of China; 2 Jiangsu Co-Innovation Center for Prevention and Control of Important Animal Infectious Diseases and Zoonoses, Yangzhou University, Yangzhou, Jiangsu 225009 People’s Republic of China; 3 Department of Animal Science, Washington State University, Pullman, WA 99163 USA; 4 Northern Jiangsu People’s Hospital Affiliated to Yangzhou University, Yangzhou, Jiangsu 225009 People’s Republic of China

**Keywords:** Corticosterone-induced chronic stress, Sodium butyrate, Intestinal barrier function, Glycolipid metabolism disorder

## Abstract

In intensive livestock and poultry farming, chronic stress poses a significant threat to animal health, primarily through its impact on butyric acid levels and intestinal microbiota balance. In our previous study, we found that the intestinal butyric acid level was significantly reduced in chronic stress models, suggesting that butyrate supplementation should be an effective anti-chronic stress strategy. Therefore, in this study, we aimed to investigate whether sodium butyrate (NaBu) prevents chronic stress-induced lipid metabolism disorders and intestinal injury by reducing intestinal damage and remodeling the microbiota. In vitro models, IEC-6 cells, a small intestinal epithelial cell line, were divided into three groups, including control (CON), corticosterone (CORT, 400 μM) and CORT + NaBu (400 μM CORT + 1 mM NaBu). In vivo models, sixty mice were divided into CON, CORT (intraperitoneal injection of 20 mg/kg CORT) and CORT + NaBu (intraperitoneal injection of 20 mg/kg CORT + intragastric gavage with 200 mg/kg NaBu). The results showed that NaBu effectively reversed CORT-induced IEC-6 migration inhibition, inhibited apoptosis, and restored barrier function, which was associated with downregulated IL-12 and upregulated LRP5 expression. In addition, NaBu administration prevented anorexia, promoted body weight gain, and improved lipid metabolism in a corticosterone-induced chronic stress model in mice, as evidenced by a significant reduction of serum alkaline phosphatase (AKP) and Low-density lipoprotein cholesterol (LDL-C). Histopathological staining and scanning electron microscopy analyses revealed that NaBu treatment restored the normal villi morphology of the small intestinal villi and mitigated epithelial cell damage caused by CORT. 16S rRNA sequencing demonstrated that NaBu facilitated intestinal microbiota remodeling, marked by significant increases in *Desulfobacterota* and *Bacillus* abundance and decreases in *Proteobacteria* and *Enterorhabdus* abundances. Furthermore, analysis suggests that CORT-induced chronic stress may disrupt the cecal microbiota-metabolite axis and lead to intestinal injury and metabolic abnormalitiesbased on previous metabolic evidence and current results of microbial imbalance and downstream metabolic/intestinal abnormalities. These findings indicate that NaBu exerts a protective role in the prevention of chronic stress-related disorders, providing a potential prophylactic strategy for animals at high risk of stress exposure.

## Highlights

➢ NaBu prevents CORT-induced anxiety-like behavior (NSFT) and anhedonia (SPT) in mice, alongside improving glycolipid metabolism and intestinal barrier function.

➢ NaBu effectively reversed migration inhibition, inhibited apoptosis, and restored barrier function in CORT-treated IEC-6 cells.

➢ NaBu facilitated intestinal microbiota remodeling and restored intestinal morphology, mitigating epithelial cell damage caused by CORT.

➢ NaBu demonstrates therapeutic potential for stress-related diseases via modulating the gut-microbiota-metabolite axis in both human and animal health applications.

## Introduction

Stress is a common psychophysiological response of the body in the face of unpleasant, demanding, or difficult situations, known as stressors (Yaribeygi et al. 2017; Schneiderman et al. 2005). Acute stress, an “emergency mobilization” mechanism for the body to cope with sudden stimuli, has the characteristics of rapid start, high intensity, and short duration, and is an adaptive short-term response. In contrast, chronic stress is a persistent or prolonged pressure known to be related to maladaptive responses, resulting in harmful effects on vital organs such as the brain, heart, or liver, in various aspects, which alter physiological and behavioral responses as well as lead to a frank disease status (Wang et al. 2024a). Chronic stress is characterized by the activation of the hypothalamic–pituitary–adrenal (HPA) axis, elevating serum ACTH and cortisol levels, two significant biomarkers for stress and chemicals that prepare the body for a fight or flight reaction. In addition, compared to acute stress, chronic stress has more serious health consequences and leads to latent viral activation, ultimately affecting the immune system(McEwen 2012). In livestock and poultry, chronic stress is one of the most stressful events in the life with harmful consequences for health, productivity and product quality due to their rapid metabolic rate and growth, high level of production, impairing the hypothalamic feeding center, and reducing feed intake. Concurrently, chronic stress causes intestinal villi atrophy and compromises barrier function, disrupting nutrient absorption and immune responses, which in turn leads to diminished production performance (Quinteiro-Filho et al. 2010). Consequently, chronic stress can compromise intestinal homeostasis by elevating cortisol levels, representing a significant challenge in livestock and poultry production. Therefore, it is imperative to identify effective strategies to combat chronic stress by restoring intestinal homeostasis in both human and animal health.

The gut microbiota represents a complex and dynamic ecosystem contributing essential functions to its host, including metabolism regulation, immune system development, nutrient absorption and pathogen colonization prevention (Nunez et al. 2025), while alterations in intestinal microbiota diversity, composition, and function are intricately linked to numerous diseases (Jiang et al. 2023; Proctor et al. 2023), such as chronic stress-related diseases (Qing et al. 2025; Costa and Lucarini 2025). So far, a growing number of evidence has demonstrated that the abundance and diversity of intestinal microbiota in hosts are highly susceptible to various stresses, underscoring the increasing interest in the role of gut microbiota in chronic stress-related diseases. Therefore, it is an effective strategy to combat chronic stress-related diseases by regulating the composition of the host intestinal flora. Furthermore, in our previous study, we revealed the disrupted lipid metabolism, impaired intestinal mucosal barrier integrity, and microbiota alterations in a CORT-induced chronic stress model in yellow-feather broilers (Li et al. 2024), which also implies that chronic stress-mediated impairments are due to the intestinal flora dysregulation. Metabolomic analyses further indicated a strong correlation between intestinal microbiota and their metabolites, which demonstrated that Chronic Corticosterone-Induced Stress (CCIS) significantly reduced the levels of butyrate, a common short-chain fatty acid (SCFA) known to support gut health and immune modulation. However, they warrant further investigation for their potential application in functional foods targeting gut health.

Butyrate is naturally produced in the large intestine, plays a crucial role in livestock nutrition, with numerous studies consistently demonstrating its significant positive effects on growth, intestinal health, and modulation of the gut microbiota (Watanabe et al. 2023; Zhang et al. 2023; Dengler et al. 2021, 2015). As a mineral form of SCFAs, butyrate is considered a promising feed supplement due to its beneficial effects on intestinal health (MáTIS et al. 2022; Bełdowska et al. 2024). Butyrate supplementation has gained considerable attention for its potential benefits in livestock, particularly concerning intestinal health and growth performance. For instance, dietary butyrate supplementation improves intestinal barrier function in piglets and alleviates high-fat diet-induced intestinal inflammation in broilers (Ding et al. 2010; Gao et al. 2022). In addition, recent studies have shown that butyrate has multiple effects, including supporting intestinal development, regulating immune responses, maintaining gut barrier integrity, and promoting gut microbiota balance in calves (He and Dong 2023). In pigs, it also showed that in the LPS-induced inflammatory model, butyrate supplementation significantly improved the gut morphology and reduced inflammation, by increasing beneficial bacteria while reducing harmful ones, and thus protected gut health during stress and infection (Han et al. 2022). In sheep and goats, sodium butyrate supplementation significantly promoted intestinal barrier and modulated the gut microbiota (Sun et al. 2024). These above results showed that sodium butyrate can be used to develop functional food products for better gut health and overall well-being. However, although extensive evidence supported butyrate's therapeutic potential, whether oral supplementation of sodium butyrate prevents lipid metabolism disorders and intestinal injury by modulating immunity, intestinal barrier functions, and gut microbiota in a CORT-induced chronic stress model is largely unknown.

While butyrate’s benefits for gut health are well-documented, most prior work focused on single outcomes (e.g., barrier function or microbiota) in non-CORT stress models (e.g., restraint stress, LPS challenge). Few studies have systematically integrated behavioral, metabolic, intestinal morphological, and microbiota data in a corticosterone-induced chronic stress model. Therefore, the present study aimed to determine the improvement effect of sodium butyrate on lipid metabolism disorders and intestinal injury, both in vitro (chronic corticosterone-induced stress in the IEC-6 cell model) and in vivo (mice model of chronic corticosterone-induced stress), especially focused on modulating immunity, intestinal barrier functions, and gut microbiota. The current results will explore the potential application of sodium butyrate as an environmental-friendly antibiotic substitute for enhancing intestinal health and mitigating stress responses, so as to provide a theoretical foundation for developing preventive and therapeutic strategies against chronic stress in both human and animals.

## Results

### Oral supplementation with NaBu significantly reversed the dietary behavior disorders in a corticosterone-induced chronic stress model in mice

**Fig. 1:**
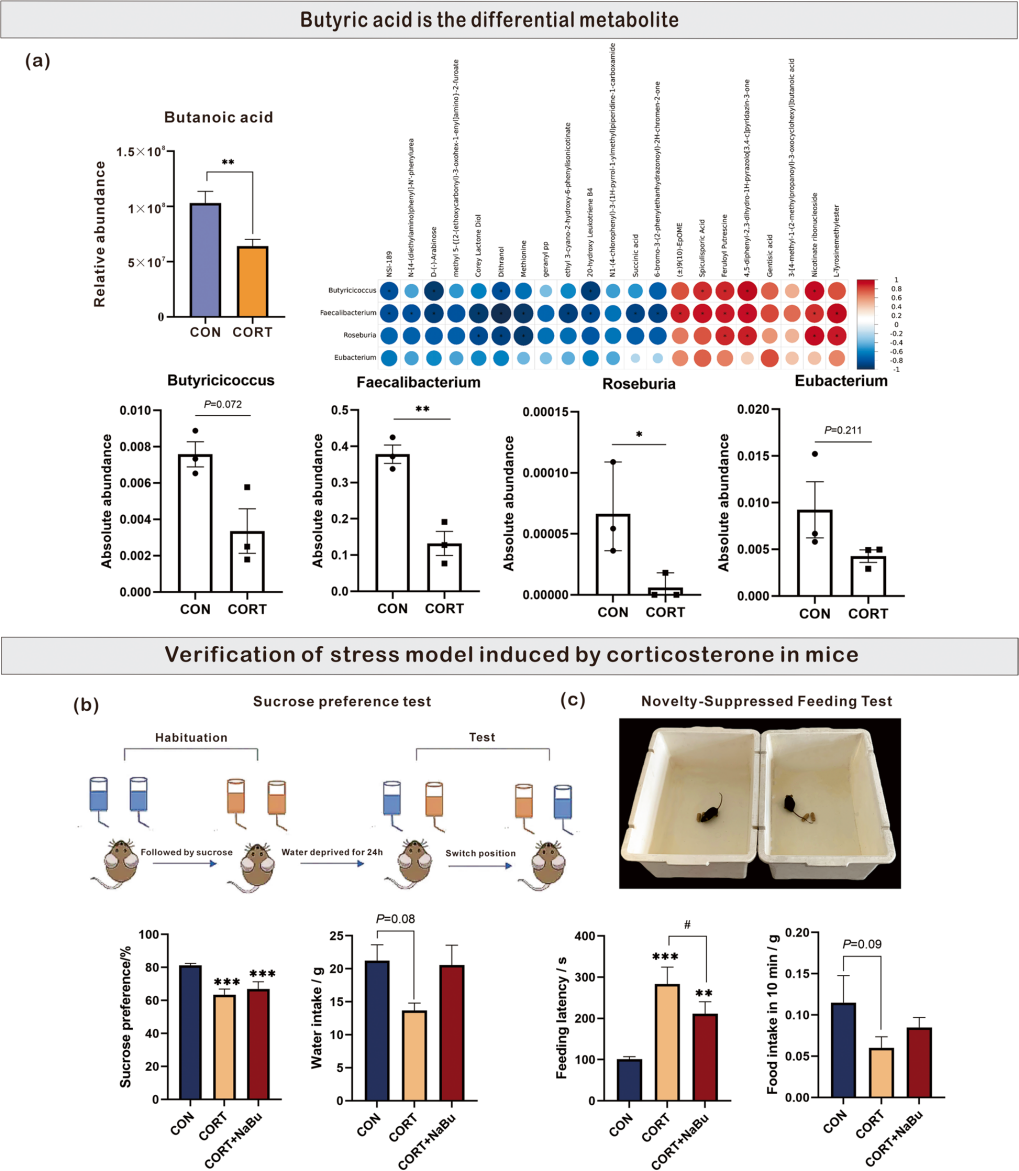
Oral supplementation with NaBu significantly reversed the dietary behavior disorders in CORT-induced chronic stress mice model. **a** Reduced butanoic acid in the intestine is responsible for CORT-induced chronic stress. **b** The sucrose preference and water intake based on the Sucrose Preference Test. **c** The feeding latency and food intake based on the Novelty-Suppressed Feeding Test. All values are represented as the means ± SEM, *n* = 10/group. ^*^P < 0.05, ^**^P < 0.01, ^***^P < 0.001 between CON and CORT groups, ^#^*P* < 0.05 between CORT and CORT + NaBu groups

In our previous study, we established a corticosterone-induced chronic stress model in yellow-feather broiler, and demonstrated that CORT treatment significantly reduced the content of butanoic acid in cecal contents through microbiological and metabolomic analysis, suggesting that as an important reduced metabolite in cecal contents, butanoic acid may be closely related to stress response (Fig. 1a). In addition, subsequent correlation analysis identified four core butanoic acid-producing bacterial genera with high expression abundance: *Faecalibacterium*, *Roseburia*, *Butyricicoccus*, and *Eubacterium*. Quantitative assessment demonstrated that the abundance of the above four core bacteria, particularly *Faecalibacterium* and *Roseburia*, was significantly reduced in the CORT group compared to CON group (Fig. 1a). These findings suggest that reduced intestinal microbial metabolite butanoic acid is closely related to CORT-induced chronic stress, and oral supplementation with NaBu may exert protective effects against chronic stress.

To determine the reverse effect of oral supplementation with NaBu on the dietary behavior disorders in corticosterone-induced chronic stress mice, 60 healthy male C57BL/6 mice were randomly divided into three groups: 1) The control group (CON) received daily oral administration of distilled deionized water (ddH_2_O) throughout the experiment, followed by intraperitoneal injection of normal saline for 2 weeks beginning at the third week. 2) The CORT group (CORT) was given daily oral ddH_2_O and intraperitoneal injection of corticosterone (CORT, 20 mg/kg body weight) daily for the same 2-week period. 3)The CORT and NaBu combined group (CORT + NaBu) received daily oral NaBu (200 mg/kg body weight) during the experimental period, and intraperitoneal CORT injection (20 mg/kg body weight) was also conducted for 2 weeks starting from the third week. One week post-treatment, both the Sucrose Preference Test and Novelty-Suppressed Feeding Test were used to assess behavior disorders (The schematic diagram of the entire experimental routes is shown in Fig. 1b-c). The sucrose preference of CORT group was significantly lower than that of CON group (*P* < 0.001) (Fig. 1b), but there was no statistical difference between CORT + NaBu group and CORT group, only showing a recovery trend. In the Novelty-Suppressed Feeding Test, CORT-treated mice exhibited significantly prolonged feeding latency (*P* < 0.001) and a decreasing trend in food intake compared to CON mice. Notably, NaBu administration significantly attenuated the above effect, demonstrating shorter feeding latency than the CORT group (*P* < 0.05) (Fig. 1c). These findings confirm the successful establishment of the chronic stress model and suggest that NaBu treatment rescues CORT-induced anxiety-like behavior but not anhedonia in mice.

### Oral supplementation with NaBu significantly prevented the body weight loss, glycolipid metabolism disorders and organ damage in a corticosterone-induced chronic stress model in mice.

**Fig. 2:**
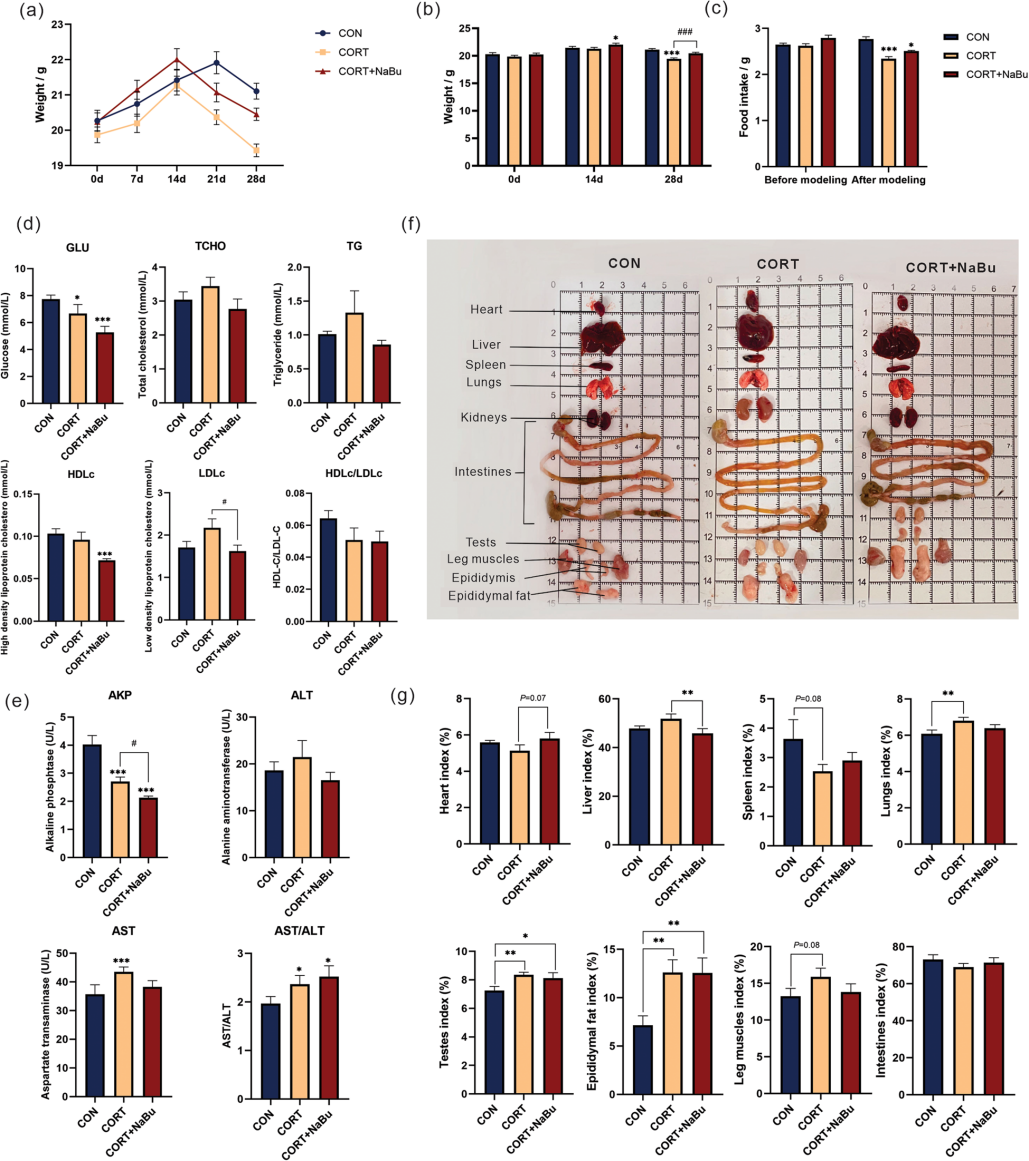
NaBu supplementation ameliorates weight loss and mitigates glycolipid metabolism dysregulation in CORT-induced chronic stress mice. **a** Line chart of body weight change within 4 weeks. **b** Body weight after treatment for 0, 14 and 28 days. **c** Food intake before and after modeling. **d** The serum contents of GLU, TCHO, TG, HDLc, LDLc, and the ratio of HDLc/LDLc. **e** The serum contents of AKP, ALT, AST, and the ratio of AST/ALT. **f** Organ display diagram of mice in each group. **g** Organ index statistics. All values are presented as means ± SEM, *n* = 20/group. ^*^P < 0.05, ^**^P < 0.01, ^***^P < 0.001 between CON and CORT groups, ^#^*P* < 0.05 between CORT and CORT + NaBu groups

In order to evaluate the effect of NaBu on growth performance in corticosterone-induced chronic stress model mice, body weight changes and food intake among different treatment groups were systematically determined. As illustrated in Fig. 2a-b, both CORT and CORT + NaBu groups exhibited rapid weight reduction post-modeling compared to CON mice. By day 28, the CORT group demonstrated significantly lower final body weight than CON mice (*P* < 0.001), while the CORT + NaBu mice showed marked weight recovery compared to CORT mice (*P* < 0.001), accompanied by moderate increases in food intake (Fig. 2c).

Blood biochemical analysis demonstrated significant alterations in the CORT group compared to CON group: decreased serum glucose (GLU) and alkaline phosphatase (AKP) (*P* < 0.05 and *P* < 0.01, respectively), alongside elevated aspartate aminotransferase (AST) and the ratio of AST/ALT (*P* < 0.01 and *P* < 0.05, respectively). NaBu intervention significantly reduced AKP and low-density lipoprotein cholesterol (LDLc) levels compared to the CORT group (*P* < 0.05).

Organ index analysis revealed significant increases in lung, testis, and testicular fat index in the CORT group compared to CON group (*P* < 0.01), with a decreased trend of kidney index (*P* = 0.08) and an increased trend of leg muscle index (*P* = 0.08). Notably, the CORT + NaBu group exhibited significant elevation in testicular fat index (*P* < 0.01) and reduced liver index (*P* < 0.01) compared to the CORT group (Fig. 2d-g). To some extent, these findings collectively indicate that NaBu supplementation ameliorates weight loss and mitigates glycolipid metabolism dysregulation in CORT-induced chronic stress mice.

### NaBu supplementation significantly prevented the intestinal inflammation and the damage to the barrier function in corticosterone-induced chronic stress model mice

In order to determine the alleviation effect of NaBu supplementation on the intestinal function, the morphological structure, inflammation and the barrier function of small intestine were examined. Histological examination revealed marked morphological differences between experimental groups. Compared with CON mice, the CORT-treated group exhibited significant intestinal alterations, including shortened, finger-shaped villi in the duodenum, jejunum, and ileum, accompanied by mucosal atrophy and villus detachment. In contrast, CON + NaBu group maintained normal architecture, characterized by slender, densely packed villi with Eosin dye solution-stained Paneth cells at crypt bases. Moreover, the surface of cecum is flat without villi, and its mucosa is mainly composed of crypts. Cecal morphology showed CORT mice showed mice disordered crypt shape, increased crypt depth, as well as atrophied and thinned mucosa, while CON and CORT + NaBu groups preserved regular crypt structure.

**Fig. 3:**
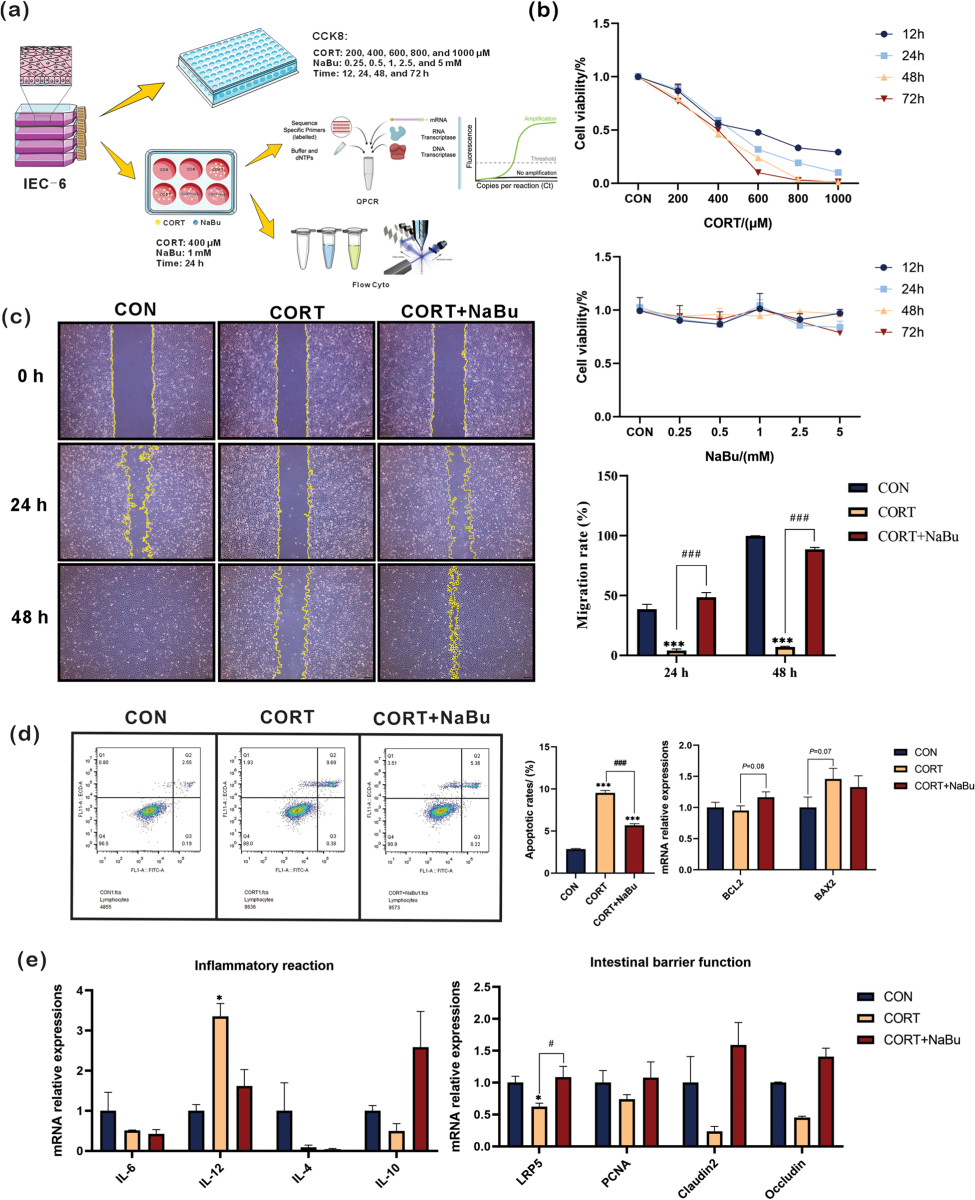
NaBu supplementation rescued the intestinal inflammation and impairment of barrier function caused by corticosterone-induced chronic stress. **a** Effects of corticosterone-induced chronic stress on the morphology of the duodenum, jejunum, ileum, and cecum after H&E staining (200 ×), along with statistical results for villus height (VH), crypt depth (CD), and the VH/CD ratio in these intestinal segments; **b** Scanning electron microscope observations of the intestinal mucosa (300 ×); **c** Expression of genes related to inflammatory responses and intestinal barrier function. All values are presented as means ± SEM, *n* = 10/group. ^*^P < 0.05, ^**^P < 0.01, ^***^P < 0.001 between CON and CORT groups, ^#^*P* < 0.05, ^##^*P* < 0.01 and ^###^*P* < 0.001 between CORT and CORT + NaBu groups

Quantitative analysis demonstrated significant reductions in duodenal villus height (*P* < 0.001) and ileal villus-to-crypt ratio (*P* < 0.05), alongside increased duodenal crypt depth (*P* < 0.01) in the CORT group compared to CON group. Notably, compared with CORT group, NaBu administration effectively reversed the above pathological changes of duodenal villus height, crypt depth, and the villus-to-crypt ratio (*P* < 0.001) (Fig. 3a).

Scanning electron microscopy of the ileal terminal mucosa showed that, compared to the CON group, the intestinal villi in the CORT group were severely damaged. The villi surface appeared rough, some were locally broken, and their tips were severely damaged. In contrast, NaBu intervention significantly improved the structural defects of the ileal villi compared with the CORT group. The villi exhibited a broad, leaf-like shape with a smooth surface, intact structure, and plump appearance (Fig. 3b). These results indicate that NaBu can significantly reverse the impairment of intestinal development caused by chronic stress in mice and exert a protective effect on intestinal villi.

At the molecular level, NaBu treatment significantly regulated the inflammatory response in the ileum compared with CORT mice. Specifically, NaBu suppressed the expression of pro-inflammatory cytokines (IL-12, *P* < 0.01; TNF-α, *P* < 0.05; IL-4, *P* < 0.001) while upregulating the expression of anti-inflammatory genes (IL-10 and IL-6, *P* < 0.001). Consistent anti-inflammatory effects were also observed in the duodenal and cecal tissues, the expression of IL-4 and COX2 showed decreasing trends, whereas the levels of IL-10 and TGF-β exhibited increasing trends (Fig. 3c).

Furthermore, quantitative analysis revealed that NaBu supplementation significantly upregulated the expression of tight junction proteins in the CORT + NaBu group compared with CORT-treated mice. Specifically, Claudin mRNA expression was markedly increased in the duodenum (*P* < 0.05), jejunum (*P* < 0.001), and ileum (*P* < 0.001). Similarly, zonula occludens-1 (ZO-1) expression was significantly elevated in both the ileum and cecum (*P* < 0.001). Additionally, we observed increasing trends in the expression of other intestinal barrier markers, including Occludin in the duodenum, leucine-rich repeat-containing G-protein coupled receptor 5 (Lgr5) in the jejunum, and mucin 2 (MUC2) in both the ileum and cecum.

### Protective effect of NaBu on intestinal epithelial cells (IEC-6) under corticosterone-induced chronic stress

**Fig. 4:**
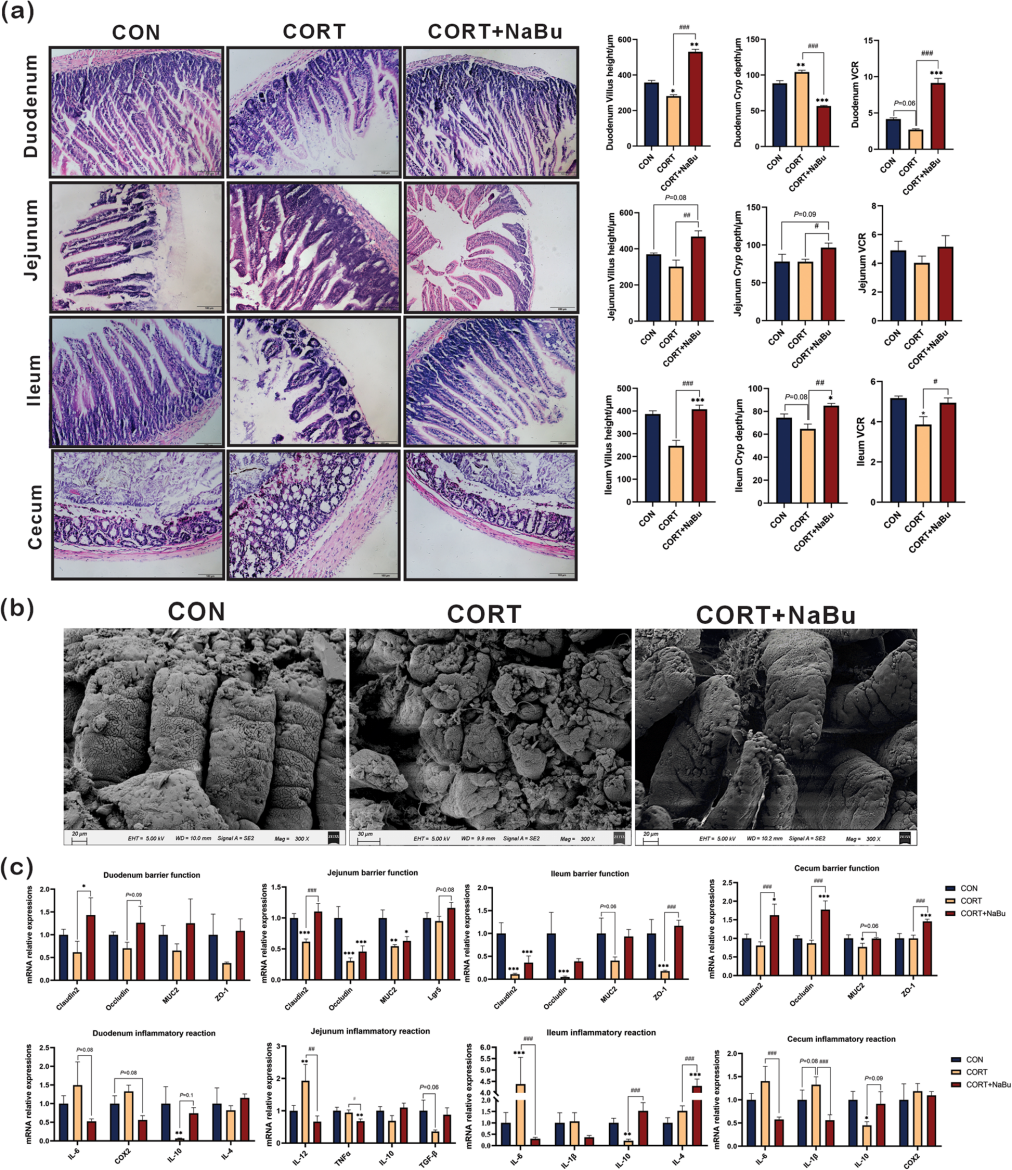
Protective effect of NaBu on IEC-6 under CORT-induced chronic stress. **a** Schematic diagram of in vitro experiment; **b** Cell activity detected by CCK-8 assay; **c** Cell mobility detected by scratch test; **d** Cell apoptosis detected by flow cytometry; **e** mRNA expression of inflammation and cell barrier-related genes. All values are presented as means ± SEM, *n* = 8/group. ^*^P < 0.05, ^***^P < 0.001 between CON and CORT groups; ^###^*P* < 0.001 between CORT and CORT + NaBu groups

In order to investigate whether NaBu supplementation has protective effects in vitro model and to determine the cellular mechanisms underlying, in vitro experiment using IEC-6 was conducted. Cell viability was assessed via CCK-8 assay following treatment with varying concentrations of CORT (200–1000 μM) and NaBu (0.25–5 mM) at different time points (12–72 h). As illustrated in Fig. 4a, CORT treatment dose-dependently inhibited IEC-6 cell viability. A concentration of 400 μM CORT reduced cell viability to approximately 50%, with consistent effects across different time points, therefore, 400 μM CORT treatment for 24 h was chosen as optimal conditions to establish chronic stress modeling. In addition, NaBu treatment demonstrated minimal cytotoxicity within the concentration range of 0.25–5 mM. Based on previous studies demonstrating enhanced intestinal barrier function at concentrations of ≤ 2 mM (Dengler et al. 2015), 1 mM NaBu and a 24-h treatment duration was selected for subsequent experiments (Fig. 4b).

Cell migration assays demonstrated that the CORT-treated group exhibited significant impairment in cell migration ability compared with the CON group at both 24 and 48 h (both *P* < 0.001). Notably, treatment with CORT + NaBu significantly reversed this CORT-induced impairment in cell migration, which indicates that NaBu exerts a protective effect against stress-induced deficits in cell migration (Fig. 4c). Apoptosis analysis showed that the apoptosis rate of CORT-treated cells was significantly higher than that of CON cells (*P* < 0.001), and this elevation was substantially mitigated by CORT + NaBu treatment (*P* < 0.001). Further molecular analysis revealed a trend toward increased BAX2 expression in the CORT group (*P* = 0.08) and a trend toward elevated BCL2 expression in CORT + NaBu cells (*P* = 0.07). These findings indicate that NaBu has the potential to prevent the apoptosis caused by CORT-induced chronic stress (Fig. 4d).

Moreover, CORT treatment significantly upregulated IL-12 mRNA expression (*P* < 0.05) while downregulated LRP5 mRNA (*P* < 0.05) compared to CON group. Furthermore, NaBu administration effectively reversed these alterations (*P* < 0.05) compared with CORT group, consistent with its anti-inflammatory and mucosal barrier protection properties against chronic stress (Fig. 4e).

### NaBu supplementation significantly reshaped cecal microbiota in corticosterone-induced chronic stress model mice

**Fig. 5:**
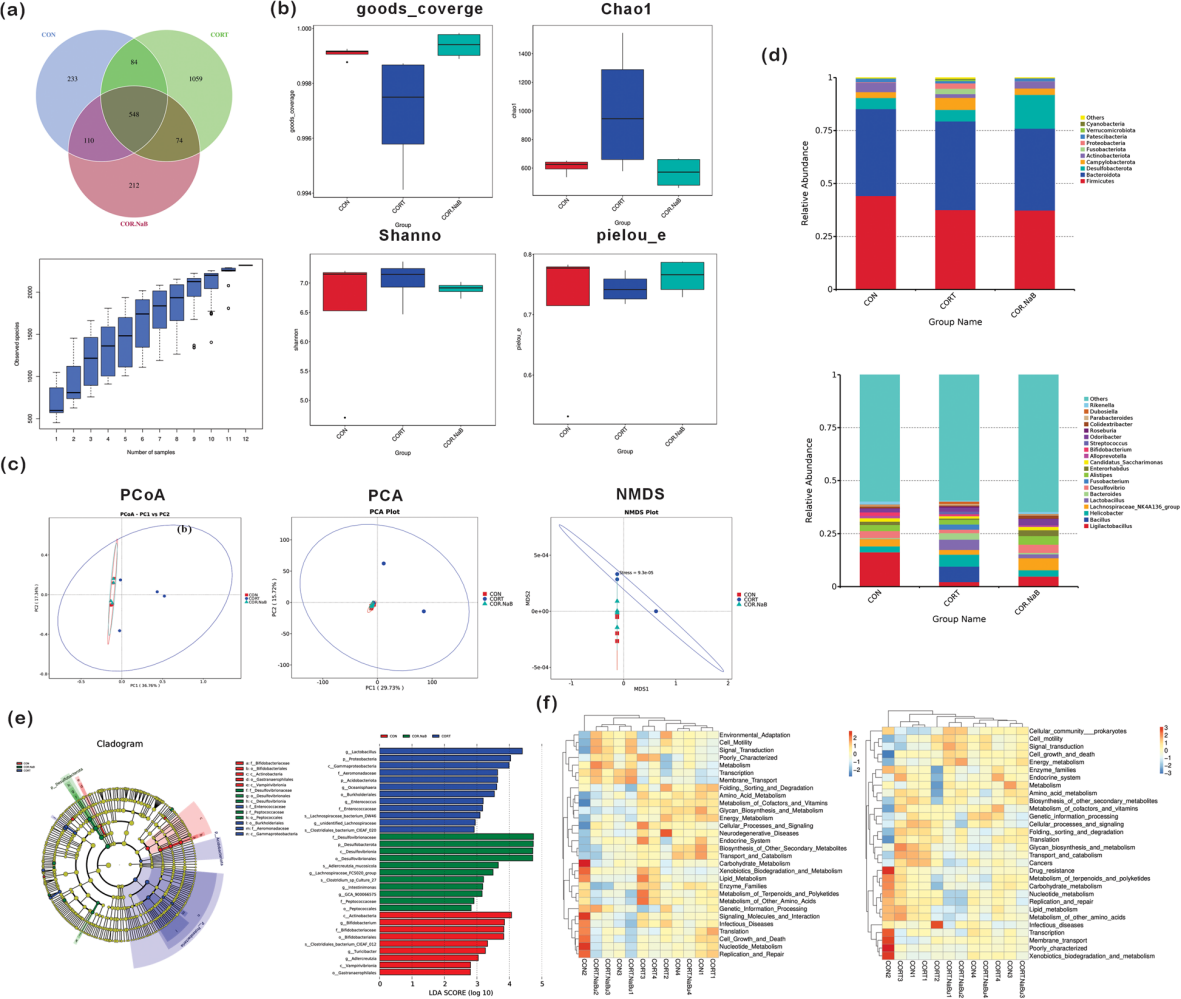
Functional changes in cecal microbiota by 16S rRNA sequencing after CORT exposure and treatment with NaBu in mice. **a** The OTUs level Venn diagram and rarefaction curve. **b** Alpha diversity of the cecum microbiota, including the Goods coverage index, Chao1 index, Shannon index, Pielou's evenness index (pielou_e), and phylogenetic diversity (PD). **c** Principal component analysis (PCA), principal coordinates analysis (PCoA), and non-metric multidimensional scaling (NMDS) based on unweighted UniFrac distances. **d** The composition of the cecum microbiota at the phylum and genus levels. **e** Linear discriminant analysis effect size (LEfSe). **f** KEGG metabolic pathway heatmap

Following data preprocessing, which included deduplication and noise reduction, sequence clustering and species annotation were conducted based on a 100% similarity threshold. This analysis yielded a total of 2,320 operational taxonomic units (OTUs) across all sample groups. Venn diagram analysis revealed distinct OTU distribution patterns across groups. The CON group harbored 233 unique OTUs, the CORT group contained 1,059 unique OTUs, while the CORT + NaBu group had 212 unique OTUs, and a total of 548 OTUs were shared among all three groups (Fig. 5a). Species accumulation curves exhibited a clear stabilization trend as the sample size increased, indicating adequate sampling depth. This observation confirms that the microbial diversity in the samples was sufficiently represented, providing a reliable foundation for subsequent analyses.

To comprehensively evaluate changes in the cecal microbial community, we employed multiple α-diversity indices: Chao1 for estimating species richness, Shannon for assessing community diversity, Pielou's evenness index for measuring community uniformity, and Goods_coverage for evaluating sequencing depth. All groups exhibited Goods_coverage values exceeding 0.9, indicating sufficient sequencing depth and reliable results. Comparative analysis revealed significant alterations in CORT-treated mice, which were characterized by increased Chao1 and Shannon indices but a decreased Pielou's evenness index compared with the CON and CORT + NaBu groups. These findings suggest that chronic stress increases microbial richness and diversity while reducing community evenness in the cecum, whereas NaBu treatment exerts limited effects on restoring these parameters (Fig. 5b).

1. β diversity analysis

To evaluate β-diversity patterns, we employed three complementary multivariate analysis approaches: Principal Coordinates Analysis (PCoA), Principal Component Analysis (PCA), and Non-metric Multidimensional Scaling (NMDS). Consistent patterns emerged across all methods, with samples from the CON and CORT + NaBu groups clustered closely together, while CORT samples formed distinct, separate clusters (Fig. 5c). These results demonstrate that chronic stress significantly alters the composition and structure of the cecal microbiota. Notably, NaBu treatment effectively shifted the microbial community profiles toward CON-like patterns, indicating its potential to mitigate chronic stress-induced dysbiosis in the cecal microbiota.

(2) Analysis of the composition and differences of cecum microbiota at the phylum and genus levels

Analysis of the microbial community at the phylum level identified nine predominant phyla, namely *Firmicutes*, *Bacteroidota*, *Desulfobacterota*, *Campylobacterota*, *Actinobacteriota*, *Fusobacteriota*, *Proteobacteria*, *Verrucomicrobiota*, and *Cyanobacteria*. Among these, *Firmicutes* (CON: 0.4423 ± 0.0834; CORT: 0.3757 ± 0.0365; CORT + NaBu: 0.3740 ± 0.0259) and *Bacteroidota* (CON: 0.4099 ± 0.0770; CORT: 0.4186 ± 0.0205; CORT + NaBu: 0.3857 ± 0.0499)stood out as the dominant phyla, each accounting for approximately 40% of the total microbial community, and no significant differences were observed in their relative abundances across groups. *Desulfobacterota* (CON: 0.0523 ± 0.0105; CORT: 0.0544 ± 0.0145; CORT + NaBu: 0.1598 ± 0.0161)accounted for approximately 8% of the total microbial community, and its relative abundance was significantly enriched in the CORT + NaBu group compared with both the CON and CORT groups (*P* < 0.01). In contrast, Fusobacteriota (CON: 0; CORT: 0.0064 ± 0.0027; CORT + NaBu: 0)exhibited an increasing trend in CORT-treated mice, though this change did not reach statistical significance. Notably, the relative abundance of Proteobacteria (CON: 0.0031 ± 0.0003; CORT: 0.0253 ± 0.0130; CORT + NaBu: 0.0011 ± 0.0000)was significantly higher in De Vos et al. (2022) the CORT group than in the CON and CORT + NaBu groups (*P* < 0.05).

At the genus level, the microbial community was dominated by *Ligilactobacillus*, *Bacillus*, *Helicobacter*, *Lachnospiraceae_NK4A136_group*, *Lactobacillus*, *Bacteroides*, *Desulfovibrio*, *Fusobacterium*, *Alistipes*, and *Enterorhabdus*. Comparative analysis revealed several significant alterations: *Bacillus* (CON: 0; CORT: 0.0729 ± 0.0465; CORT + NaBu: 0) abundance exhibited an increasing trend, while *Lactobacillus* level (CON: 0.0008 ± 0.0006; CORT: 0.0486 ± 0.0154; CORT + NaBu: 0.0176 ± 0.0055) was significantly higher in the CORT group compared to CON group (*P* < 0.01). NaBu intervention significantly reduced *Lactobacillus* abundance compared to the CORT group (*P* < 0.05), though its level remained elevated compared to CON group (*P* < 0.05). Furthermore, NaBu treatment significantly decreased *Enterorhabdus* abundance (CON: 0.0158 ± 0.0047; CORT: 0.0064 ± 0.0027; CORT + NaBu: 0.0278 ± 0.0074) in the CORT + NaBu group compared with CORT-treated mice (*P* < 0.01) (Fig. 5d).

(3) LEfSe

Linear discriminant analysis Effect Size (LEfSe) analysis revealed distinct microbial signatures across different groups. The CON group exhibited nine characteristic taxa, predominantly within the Actinobacteria phylum, including *Bifidobacterium* (class: *Actinobacteria*; family: *Bifidobacteriaceae*; order: *Bifidobacteriales*), *Turicibacter*, *Adlercreutzia*, and *Clostridiales_bacterium_CIEAF_012*, along with *Vampirivibrionia* and *Gastranaerophilales*.

CORT mice demonstrated twelve signature taxa, mainly characterized by *Lactobacillus*, Proteobacteria phylum members (*Gammaproteobacteria*, *Aeromonadaceae*, *Oceanisphaera*), and *Enterococcus* (family: *Enterococcaceae*), complemented by *Lachnospiraceae*-related species (*bacterium_DW46*, *unidentified_Lachnospiraceae*, *bacterium_CIEAF_020*) and *Acidobacteriota*.

The CORT + NaBu group showed eleven distinctive taxa, dominated by sulfate-reducing bacteria (*Desulfovibrionaceae*, *Desulfobacterota*, *Desulfovibrionia*, *Desulfovibrionales*) and *Lachnospiraceae_FCS020_group*, accompanied by *Peptococcaceae*, *Adlercreutzia_mucosicola*, *GCA_900066575*, *Peptococcales*, *Intestinimonas*, and *Clostridium_sp_Culture_27* (Fig. 5e).

(4) Functional changes in cecal microbiota after CORT exposure and treatment with NaBu in mice

Functional prediction of cecal microbiota was performed using PICRUSt and Tax4Fun algorithms, with results presented in Fig. 5f. PICRUSt analysis revealed distinct functional profiles across experimental groups. CON samples exhibited significant enrichment in core metabolic processes, including carbohydrate metabolism, nucleotide metabolism, and DNA replication/repair, along with signal molecule interactions. CORT mice showed predominant enrichment in disease-related pathways (Neurodegenerative diseases, Endocrine system) and specialized metabolic functions (lipid metabolism, terpenoid/polyketide metabolism, and alternative amino acid metabolism). CORT + NaBu samples demonstrated the enhanced activity in fundamental cellular processes, particularly metabolism, membrane transport, and genetic information processing.

**Fig. 6:**
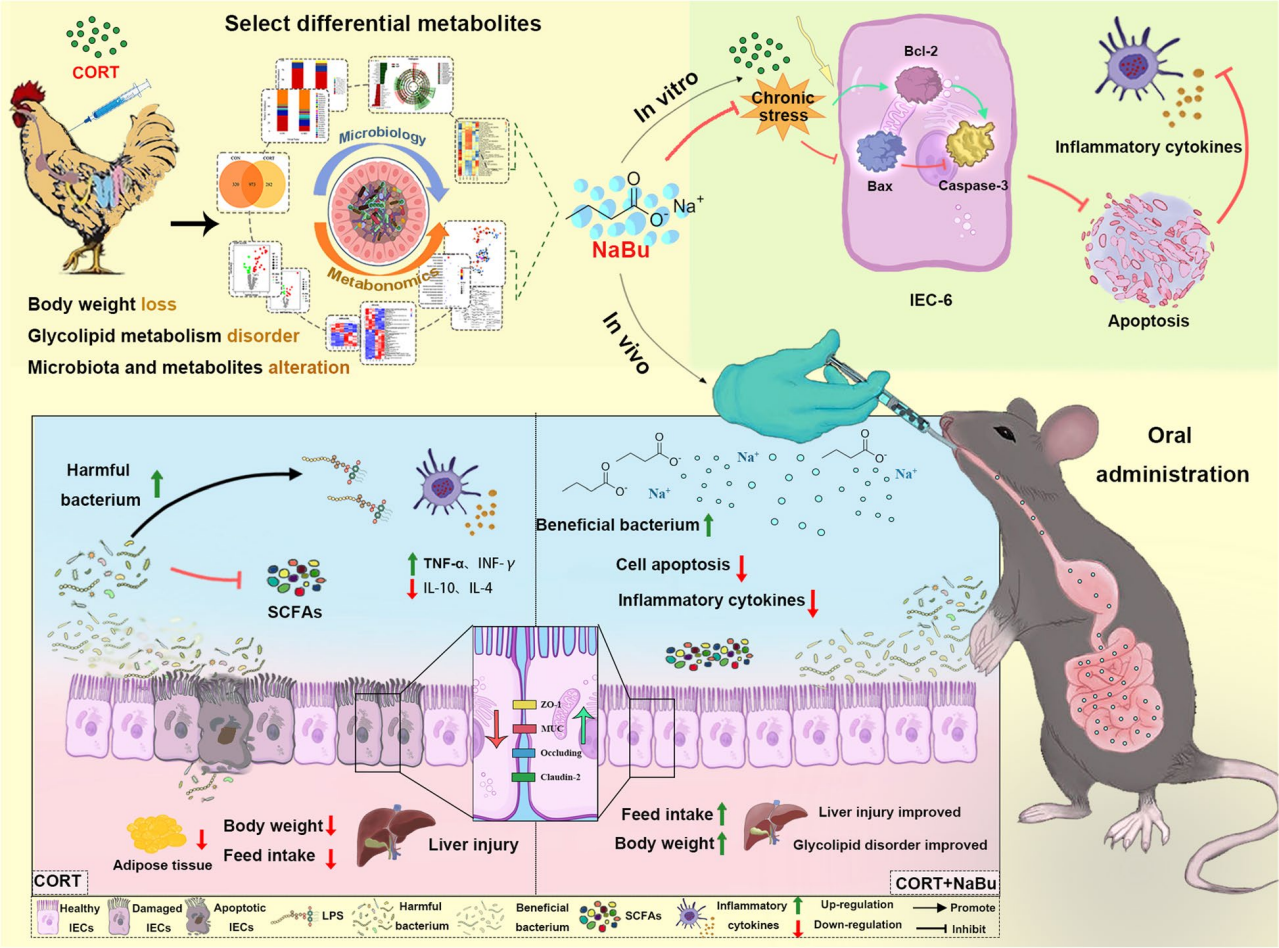
Summary of sodium butyrate, an intestinal metabolite, interacts with the microbiota to alleviate the damage caused by chronic stress to host growth and development, glycolipid metabolism, intestinal morphology, and the intestinal mucosal barrier

Complementary Tax4Fun analysis identified additional functional distinctions. CON group microbiota were primarily associated with xenobiotic processing (Drug resistance, Xenobiotics biodegradation), transcriptional regulation, and carbohydrate metabolism. CORT group samples showed marked enrichment in pathogenic pathways (Infectious diseases, Cancers) and glycan biosynthesis/metabolism. In contrast, CORT + NaBu group microbiota exhibited functional profiles centered on cellular signaling, energy metabolism, and genetic information processing. In a mouse model of corticosterone-induced chronic stress, oral administration of sodium butyrate prevents lipid metabolism disorders and intestinal injury by modulating immunity, intestinal barrier function, and gut microbiota, as summarized in Figure 6.

## Discussion

This study adds incremental novelty by providing an integrated, multi-level evaluation in a corticosterone-induced chronic stress model. We link behavioral outcomes with glycolipid metabolism, intestinal barrier, and cecal microbiota changes, and bridge these in vivo phenotypes with epithelial responses in CORT-exposed IEC-6 cells. Notably, our in vitro data implicate IL-12-LRP5 modulation as a potential epithelial node underlying NaBu protection.

Intestinal microbiota-derived secondary metabolites play a crucial role in modulating host cellular functions through interactions with various signaling pathways, particularly in metabolic tissues (BLüHER 2019). A meta-analysis of existing literature suggests that butyric acid, a prominent cecal microbiota metabolite, exhibits significant variations in abundance that may correlate with stress resilience in livestock (Fu et al. 2019; Feng et al. 2023). Prior studies reported NaBu’s effects on growth performance or intestinal health in stress models (Bortoluzzi et al. 2017), but not its ability to simultaneously reverse stress-induced anhedonia (Sucrose Preference Test) and anxiety-like behavior (Novelty-Suppressed Feeding Test). Our results extend these findings by linking NaBu to behavioral resilience, which aligns with the gut-microbiota-brain axis theory (Leigh et al. 2023). These findings show that the successful establishment of the chronic stress model and suggest that NaBu treatment rescues CORT-induced anxiety-like behavior but not anhedonia in mice. The inconsistency between SPT and NSFT results may be due to the different sensitivity of the two tests to stress-induced behavioral changes. SPT primarily evaluates anhedonia (emotional response), while NSFT assesses anxiety-like behavior and feeding motivation (functional response). Previous studies have shown that butyrate analogs preferentially improve anxiety-related behaviors rather than anhedonia in stress models (Wang et al. 2024b), which is consistent with our findings. Thus, NaBu effectively reverses stress-induced anxiety-like feeding behavior but has limited effects on anhedonia, highlighting the specificity of its behavioral protective effects.

Growth performance metrics provide valuable insights into livestock production efficiency. Numerous studies have demonstrated that dietary modifications, including NaBu supplementation, can significantly influence growth parameters (Zhang et al. 2022). For instance, NaBu-enriched milk enhances ADFI and ADG in calves, while improving feed conversion efficiency in weaned piglets (Tang et al. 2023). In the present experiment, following two weeks of CORT administration, a significant reduction in final body weight (FBW) was observed compared with CON mice. However, NaBu intervention led to substantial body weight recovery and increased ADFI, which aligns with previous findings. These results have confirmed the efficacy of NaBu in alleviating CORT-induced anorexia and promoting body weight gain in mice models. Blood biochemical analysis served as a critical indicator of nutritional metabolism and overall health status. Transaminase activity, particularly that of aspartate transaminase (AST), provides valuable insights into hepatic function and protein metabolism. As a sensitive marker of hepatocyte integrity, AST levels also reflect cardiac and renal function (Tang et al. 2023). In our results, it had no significant effects on serum transaminases (AST, ALT) or glucose metabolism, but NaBu supplementation significantly reduced serum LDL-C and AKP levels in CORT-treated mice, indicating improvements in lipid transport and hepatobiliary function. This may be due to the tissue-specificity of NaBu’s action: NaBu primarily acts on the intestinal tract to modulate lipid absorption (thereby reducing LDL-C) and exerts mild hepatoprotective effects via anti-inflammation (Dengler et al. 2021).

In addition, HDL-C in NaBu treatment group increased abnormally. However, in the context of chronic stress, lipid metabolism improvement is not defined by a single increase in HDL-C, but by the correction of core pathogenic abnormalities (e.g., LDL-C elevation, TC/TG disorders) and the restoration of cholesterol homeostasis. LDL-C is recognized as the primary risk factor for atherosclerotic diseases, and its reduction is the most critical indicator of lipid metabolism improvement in both clinical and experimental settings (Johnson et al. 2025). Elevated LDL-C levels are associated with dyslipidemia and increased cardiovascular risk. Our results showed a significant reduction in LDL-C following NaBu treatment, which is consistent with previous findings in silky fowl models that 500 mg/kg NaBu supplementation effectively lowered serum LDL-C levels (Miao et al. 2023). The potential reason for HDL-C reduction in the NaBu group is related to the functional remodeling of HDL-C under chronic stress: CORT-induced chronic stress impairs the antioxidant and reverse cholesterol transport functions of HDL-C (forming "dysfunctional HDL"), which loses its protective role and may even promote inflammation. Previous studies have confirmed that sustained elevation of cortisol (the main component of CORT) can significantly reduce HDL's cholesterol efflux capacity and antioxidant potential, leading to the accumulation of dysfunctional HDL in the serum (Moradi et al. 2013; Mertens et al. 2003). NaBu may regulate the expression of cholesterol metabolic enzymes (e.g., CETP, LCAT) by activating GPR41/GPR43 or inhibiting HDAC, thereby promoting the clearance of dysfunctional HDL-C and reducing its serum concentration (Eckardstein 2022; Hersberger and Eckardstein 2005; Dicks 2025). This process is not a sign of metabolic deterioration but rather a reflection of the restoration of lipid metabolism homeostasis, which is consistent with the findings in sodium butyrate-treated laying hens and hypercholesterolemic animal models (Miao et al. 2023; Schoch et al. 2022). Collectively, these findings highlight the great potential of NaBu to improve lipid metabolism in chronic stress models in mice.

The intestinal epithelial barrier depends heavily on short-chain fatty acids (SCFAs), particularly butyrate, for its maintenance and function. Butyrate, a four-carbon SCFA produced via microbial fermentation of dietary fiber, acts as the primary energy source for colonic epithelial cells, while also promoting barrier integrity and microbial homeostasis (Gao et al. 2021; Fang et al. 2024). Beyond its metabolic functions, butyrate exhibits anti-inflammatory properties and modulates mucosal immunity (Chong et al. 2022). While butyrate’s barrier-protective effects are known (Dengler et al. 2021; Gao et al. 2021), our study demonstrate that NaBu restores duodenal/jejunal villus height and crypt depth in CORT-induced stress-morphological changes not reported in LPS or restraint stress models (Han et al. 2022; Zheng et al. 2013). Histological analysis revealed significant morphological alterations in CORT-treated mice, including shortened, finger-shaped villi in the duodenal, jejunal, and ileal segments, mucosal atrophy, and villus detachment, compared to the CON group. NaBu supplementation substantially restored intestinal architecture, improving key parameters such as duodenal VH, CD, and the VCR, as well as jejunal and ileal VH and VCR. Scanning electron microscopy of the ileal mucosa provided further ultrastructural evidence of NaBu-mediated morphological preservation (Wang et al. 2021). At the molecular level, analysis demonstrated that NaBu significantly upregulated the expression of tight junction proteins, with increased levels of Lgr5, Claudin-1 and ZO-1 in duodenal, jejunal, and cecal tissues. These findings are the same as previous reports showing that NaBu has immunomodulatory effects in CORT-induced chronic stress broiler models, particularly by regulating IL-6 expression and microbiota-mediated immune responses (Bortoluzzi et al. 2017). Consistent with these observations, our results showed a marked upregulation of IL-6 and IL-10 in ileal tissue following NaBu treatment, accompanied by a significant downregulation of IL-12, TNF-α, and IL-4 across jejunal, ileal, and cecal segments. It should be noted that NaBu-induced IL-6 upregulation in the corticosterone-induced chronic stress model is a context-dependent adaptive response (Gao et al. 2021). It is critical to distinguish between the roles of IL-6 in acute and chronic inflammation. In acute stress or infection, IL-6 is rapidly secreted by macrophages/neutrophils as a pro-inflammatory cytokine to amplify the inflammatory response (Hunter and Jones 2015). However, in chronic inflammation (e.g., our 4-week corticosterone-induced stress model), sustained pro-inflammatory signaling leads to tissue damage, and IL-6 switches to an anti-inflammatory and tissue-repair role (Scheller et al. 2011). This functional switch is mediated by differences in receptor utilization (soluble IL-6R vs. membrane-bound IL-6R) and downstream signaling pathways (STAT3 vs. MAPK/NF-κB). In our study, the upregulation of IL-6 occurred in the context of chronic intestinal inflammation, and its co-expression with anti-inflammatory factors (IL-10) confirms its adaptive repair function rather than a pro-inflammatory role. Collectively, these findings demonstrate the efficacy of NaBu in mitigating chronic stress-induced intestinal inflammation and barrier dysfunction.

The biological effects of NaBu on small intestinal epithelial cells exhibit concentration-dependent characteristics. Zhu (2011) demonstrated that medium (8 mM) and high (14 mM) concentrations of NaBu significantly induced apoptosis and inhibited proliferation in IEC-6 cells, whereas low concentrations (2 mM) had minimal impact on cellular viability and apoptosis. Building on these findings, we conducted dose–response experiments using IEC-6 cells treated with varying concentrations of CORT (200–1000 μM) and NaBu (0.25–5 mM) across multiple time points (12–72 h). Based on previous evidence, we selected 1 mM NaBu as the optimal concentration for subsequent experiments investigating CORT-induced cellular responses. Scratch wound assays revealed that NaBu significantly enhanced cell migration capacity, mitigating the impairment induced by chronic stress. These results align with previous observations in porcine intestinal epithelial cells (IPEC-J2), where low-dose NaBu (0.5–1 mM) promotes proliferation and differentiation (Yang et al. 2020; Qiu et al. 2017). Flow cytometric analysis showed that the apoptosis rate of CORT-treated IEC-6 cells was significantly higher than that of CON cells, and this elevation was markedly reduced by NaBu intervention. Molecular analysis of apoptosis-related genes showed corresponding alterations in the expression patterns of BAX and BCL2, further confirming the anti-apoptotic effects of NaBu. Additionally, the protective role of NaBu against oxidative stress-induced epithelial injury was supported by its regulation of tight junction protein expression (Li et al. 2022). During the process by which sodium butyrate (NaBu) reverses corticosterone (CORT)-induced migration inhibition and apoptosis in IEC-6 cells, significant changes occur in IL-12 and LRP5. Historically, research on the effects of butyrate in intestinal epithelial cells (IECs) has primarily focused on regulating tight junction proteins (e.g., ZO-1, Claudin-1) to enhance barrier integrity (Dengler et al. 2021; Gao et al. 2021) or modulating classic apoptosis-related genes (e.g., BAX, BCL2) to suppress cell death (Yang et al. 2020; Li et al. 2022). However, limited attention has been paid to the crosstalk between pro-inflammatory cytokines and the Wnt signaling pathway in stress-induced cellular dysfunction. For instance (Yang et al. 2020), reported that butyrate regulates the apoptosis of IPEC-J2 cells through autophagic pathways but did not explore cytokine-mediated signal transduction (Yang et al. 2020). In another study (Li et al. 2022), demonstrated that NaBu protects IECs against oxidative stress via the AMPK-mitophagy pathway, with no association linked to IL-12 or LRP5 (Li et al. 2022). In contrast, our research identifies IL-12 and LRP5 as potential novel targets of NaBu in CORT-exposed IEC-6 cells. We show that CORT-induced upregulation of IL-12 and downregulation of LRP5 are closely associated with impaired cell migration and enhanced apoptosis. Co-treatment with NaBu effectively reverses these molecular alterations and promotes functional recovery-an interaction not previously described in butyrate-IEC studies.Collectively, these findings provide in vitro evidence of the protective role of NaBu against chronic stress-induced intestinal inflammation and barrier dysfunction.

To elucidate the impact of NaBu on gut microbiota composition, we performed 16S rRNA sequencing analysis of cecal contents from mice. α-Diversity analysis revealed increased microbial diversity and richness under chronic stress conditions, which may be attributed to stress-induced colonization and proliferation of pathogenic bacteria (Nhara et al. 2024). Unlike prior studies showing NaBu-induced increases in Firmicutes or Bacteroidota (He and Dong 2023; Bortoluzzi et al. 2017), we identified unique microbial signatures: NaBu specifically enriches Desulfobacterota (a butyrate-producing phylum (Sun et al. 2024)) and reduces Proteobacteria/Enterorhabdus (pro-inflammatory/stress-related taxa (Leigh et al. 2023)). LEfSe analysis further confirmed that the CORT group was enriched in pathogenic subtypes such as Gammaproteobacteria and Aeromonadaceae, while beneficial taxa (e.g., Bifidobacterium) were depleted. This pattern is consistent with Qing et al. (2025), who reported that chronic stress in mice increased microbial richness but was accompanied by the proliferation of opportunistic pathogens, leading to a functionally disrupted community. In addition, the remarkable decrease of Pielou evenness in CORT group indicates that the community is unstable, which reveals that the increase of Chao1/Shannon index is not a sign of a healthy and balanced microbial community, but is influenced by an unstable community dominated by a few pathogenic taxa. This further supports that the increased α diversity in CORT group is the pathological feature of stress-induced ecological disorder. Multivariate analysis (PCoA, PCA, and NMDS) showed distinct clustering patterns between the CORT and CON groups. Notably, NaBu intervention restored the microbial community structure to a profile resembling that of the CON group. Functional prediction further reveals NaBu shifts microbial pathways from neurodegenerative disease/lipid metabolism disorder to signal transduction/energy metabolism-providing novel insights into how NaBu modulates gut-microbiota-host crosstalk in chronic stress.

A limitation of the present study is that the in vitro corticosterone concentration was relatively high (400 μM), chosen to reduce IEC-6 viability to ~50%, which may exceed physiological stress levels and partly reflect cytotoxic/injury responses; therefore, the observed IL-12/LRP5 changes should be extrapolated to in vivo conditions with caution. In addition, we did not directly quantify microbial metabolites (e.g., butyrate and propionate) in mouse cecal contents, which limited our ability to determine whether NaBu altered SCFA production and to link microbial metabolites to downstream host responses. Future studies will employ lower, more physiologically relevant corticosterone ranges and integrate targeted SCFA measurements (and/or metabolomics) with microbiota and barrier/inflammatory indices to validate the IL-12–LRP5/Wnt axis in vivo.

## Conclusion

In conclusion, our study systematically evaluated the role of NaBu in a CORT stress model of mice, integrating the results of behavior, metabolism, intestine and microorganism. inducing significant alterations in the composition and metabolic function of the cecal microbiota, which in turn lead to impaired growth performance, metabolic dysregulation, and intestinal barrier dysfunction. NaBu emerges as a key modulator of host-microbiota interactions, exhibiting anti-inflammatory properties and promoting intestinal homeostasis. The synergistic relationship between NaBu and the intestinal microbiota represents a fundamental mechanism for mitigating chronic stress, Furthermore, the significant change of IL-12/LRP5 level mediated its protective effect on IEC-6, which revealed the possible molecular mechanism of NaBu's action, and also verified that it was a potential environment-friendly antibiotic substitute for livestock and a candidate adjuvant therapy for stress-related diseases. These findings provide a novel prophylactic strategy for stress-prone animals. Future therapeutic-oriented studies will further explore the potential of NaBu as a treatment for established chronic stress.

## Materials and methods

### Animal ethics

All procedures were approved by the Animal Ethics Committee of Yangzhou University and conducted in compliance with international guidelines for experimental animal ethics.

### Reagents and drugs

Sodium butyrate and corticosterone were obtained from GLPBIO. DMEM medium was gained from Gibco, and fetal bovine serum (FBS) was procured from Sigma. Additional reagents, including 0.25% trypsin, penicillin–streptomycin solution, phosphate-buffered saline (PBS), and dimethyl sulfoxide (DMSO), were purchased from HyClone. Total RNA isolator and Script III All-in-One RT Supermix, perfect for qPCR, were purchased from Vazyme. The CCK-8 kit, Annexin V-FITC/PI apoptosis detection kit, and bovine serum albumin (BSA) were bought from Biosharp. A cell cycle and apoptosis detection kit was performed by Biyotime. Cell culture bottles and multiwell plates were obtained from Thermo Scientific.

### Animal grouping and experimental design

In the study, sixty healthy male C57BL/6 mice (5 weeks old, 18 ± 2 g) were obtained from the Comparative Medical Center of Yangzhou University. After adapting to the environment for one week, the mice were randomly divided into three groups and the formal experiment lasted four weeks. The control group (CON) received daily oral administration of distilled deionized water (ddH_2_O) during the experimental period and intraperitoneal injection of normal saline for two weeks starting from the third week. The CORT group (CORT) was administered oral ddH_2_O daily and intraperitoneal injection of corticosterone (CORT, 20 mg/kg body weight) daily for the same two-week period. The CORT and NaBu combined group (CORT + NaBu) received daily oral NaBu (200 mg/kg body weight) during the experimental period, and intraperitoneal CORT injection (20 mg/kg body weight) was also conducted for two weeks starting from the third week. The concentration of NaBu used in this study was chosen according to previously published literature (Miao et al. 2023; Wang et al. 2021). Notably, NaBu administration was initiated 2 weeks prior to CORT injection to establish a preventive intervention model. Mice were housed under standard conditions (23 ± 2 °C) with ad libitum access to water and a standard diet (Feed composition and nutritional details are provided in Table 1). Body weight and feed intake were recorded weekly to calculate body weight gain (BWG), average daily gain (ADG), average daily feed intake (ADFI), and feed conversion rate (F/G).

**Table 1:** Chemical composition of feed ingredients and diets

Component	Proportion
Casein 80 orders	18.96%
Corn starch	32.30%
Maltodextrin (DE = 10)	13.20%
Sucrose	20.14%
Cellulose	4.74%
Soybean oil	2.37%
Lard	2.63%
Small material	5.66%
Total	100%

After four weeks, mice were fasted and weighed at 8:00 a.m.. Then, mice were euthanized by cervical dislocation, and tissues (heart, liver, spleen, kidneys, gastrocnemius muscle, testes, epididymal fat, and intestines) were collected for organ index analysis. Organ index: *n* = 20/group. The organ index is calculated as follows: Organ index (%) = [organ weight (g) ÷ body weight (g)] × 100%.

### Sucrose preference test (SPT)

Sucrose preference test (SPT) is a classic behavioral test to evaluate the lack of pleasure. its principle is a detection method designed by using rodents' preference for sweetness. After fasting for a period of time, animals are given normal drinking water and low-concentration sucrose water at the same time, and the animal's preference for sucrose water (sugar water preference index) is used as an index to judge whether animals have the depressive symptom of lack of pleasure. In the study, sucrose preference test was used to investigate the reduction in responding for positive affective stimuli in mice under the treatment of CORT, and the reverse effect of NaBu treatment. In this experiment, mice were individually housed and acclimated to cages with food and water. Firstly, mice had free access to two 30 ml bottles containing1% (w/v) sucrose solution for 24 h as an acclimation period. Then, one of the bottles was changed with tap water and left for another 24 h, the bottles were repositioned every 12 h to prevent positional bias. Following adaptation to the sucrose solution, mice were fasted for 12 h, after which each cage was provided with two 30 ml bottles, one with tap water and the other with 1% sucrose solution. The bottle placement was switched every 12 h, and the consumption of both sucrose solution and tap water was precisely measured over 24 h. Sucrose preference was determined as the percentage of 1% sucrose volume consumed over the total fluid intake volume. Sucrose preference (percentage) was calculated as the following formula. Behavioral tests (SPT): *n* = 10/group.

$$\mathrm{Sucrose}\;\mathrm{preference}\;(\%)\;=\frac{1\%\;\mathrm{Sucrose}\;\mathrm{solution}\;\mathrm{consumption}\;(\mathrm{mL})}{\mathrm{Total}\;\mathrm{consumption}\;\mathrm{of}\;1\%\;\mathrm{sucrose}\;\mathrm{solution}\;\mathrm{and}\;\mathrm{drinking}\;\mathrm{water}\;(\mathrm{mL})}\;\times\;100\%$$

### Novelty-Suppressed Feeding Test (NSFT)

Novelty-Suppressed Feeding Test (NSFT) is a behavioral experimental method commonly used to evaluate the effects of antidepressants and emotional responses in animal models. In the experiment, the degree of stress reaction and anxiety can be inferred by observing the behavior of animals eating novel food in a new environment. It is the contradiction between feeding and fear of the new environment in a novel environment when animals are hungry after fasting. Long-term administration of antidepressants can reduce the conflict reaction of animals, which shows that the incubation period of starting feeding is shortened. This model was applied after modeling and is widely used in the long-term effect evaluation of antidepressants and anti-anxiety drugs. In the study, a white opaque mouse cage with an open top was used as a test box, and the weighed feed particles were placed in the center after the inner wall was disinfected with alcohol. After a 24-h fasting period, mice were transferred into the chamber from the same corner. The time from putting the mouse into the test box to taking food for the first time was recorded, named as the food intake latency (the criterion for judging food intake is that mice start chewing food, not just smelling or fiddling with food intake latency). The test duration was set at 10 min, with mice failing to consume food within this period assigned a latency of 10 min. After 10 min of testing, the remaining food was weighed to calculate the food consumption rate. Behavioral tests (NSFT): *n* = 10/group.

$$\mathrm{Food}\;\mathrm{consumption}\;\mathrm{rate}\;(\%)\;=\;\frac{\mathrm{Initial}\;\mathrm{food}\;\mathrm{weight}\;-\mathrm{Food}\;\mathrm{weight}\;\mathrm{after}\;10\;\min}{\mathrm{Initial}\;\mathrm{food}\;\mathrm{weight}}\;\times\;100\%$$

### Detection of serum biochemical indexes

After four weeks, mice were fasted and weighed at 8:00 a.m.. Blood samples were collected from the ocular venous plexus of mice into 1.5 mL centrifuge tubes. Serum biochemistry: *n* = 20/group. Serum was separated by centrifugation (4 °C, 3000 × g, 10 min) and stored at −20 °C for subsequent biochemical analysis. Serum biochemical parameters, including glucose (Glu, F006-1-1), total cholesterol (TC, A111-1-1), triglycerides (TG, A110-1-1), high-density lipoprotein cholesterol (HDL-C, A112-1-1), low-density lipoprotein cholesterol (LDL-C, A113-1-1), total protein (TP, A045-2-2), albumin (Alb, A028-2-1), globulin (Glb, A106-1-1), alkaline phosphatase (ALP, A059-2-2), alanine aminotransferase (ALT, C009-2-1), and aspartate aminotransferase (AST, C010-2-1), were quantified by using commercial kits purchased from the Nanjing Jiancheng Institute of Bioengineering (China).

### Paraffin section of intestinal tissue, hematoxylin and eosin (H&E) staining and Periodic Acid-Schiff (PAS) staining

Intestinal segments (duodenum, jejunum, ileum, cecum) were fixed in 10% formaldehyde and subsequently processed using standard paraffin embedding techniques. Hematoxylin and eosin (H&E) staining was performed to evaluate intestinal villus morphology. Well-oriented villi and crypts were selected for morphometric analysis, including measurements of villus height (VH), villus width, and crypt depth (CD). The villus surface area (VSA) and villus height-to-crypt depth ratio (VCR) were subsequently calculated.

Jejunum sections were stained with periodic acid-Schiff (PAS). Five representative fields per section were captured at 400× magnification. Goblet cell density was determined by counting goblet cells in five fields of view per slide, standardized using a 400× micrometer, and expressed as the number of goblet cells per unit villus area. Mucus layer thickness was measured at the center of five villi per field, with two measurements taken per villus, and the average mucus layer thickness was calculated for each section.

Intestinal histology: *n* = 4/group. Mice were randomly assigned to experimental groups using a random number generator. Histological staining (H&E/PAS) and behavioral test scoring were conducted in a blinded manner-investigators were unaware of group assignments during sample processing and data recording.

### Scanning electron microscope of terminal ileum tissue

Tissue fragments at the end of ileum were separated and immediately placed on ice. The tissue was trimmed into 0.5 cm^3^ blocks and fixed in 2.5% glutaraldehyde for 1–3 h. Following fixation, the samples were washed 3–4 times with phosphate-buffered saline (PBS), each for 10–15 min. The samples were dehydrated through a graded ethanol series and then dried using a critical point dryer. Dried samples were mounted on specimen stubs with the observation surface facing upward and sputter-coated with gold using an ion sputterer. Finally, the samples were examined under a scanning electron microscope.

### RNA isolation and qRT-PCR

Total RNA was extracted from intestinal tissues and cell samples using an RNA isolator (Vazyme, R401), and RNA concentration was quantified using a NanoDrop 2000 spectrophotometer (Thermo Scientific). All primers, listed in Table 2, were synthesized by Genewiz, Inc. (Suzhou, China). Gene expression levels were normalized to the reference gene GAPDH and calculated using the 2^−∆∆CT^ method. All experiments were performed in triplicate.

**Table 2:** The primers used in the present study

Gene	Primer sequences (5’−3’)	Product size	Genbank number
IL-1β	F: AGGAGAACCAAGCAACGACA	241	NM_008361.4
	R: CTCTGCTTGTGAGGTGCTGA		
IL-6	F: TAGTCCTTCCTACCCCAATTTCC	216	NM_001314054.1
	R: TTGGTCCTTAGCCACTCCTTC		
IL-10	F: ATGCAGGACTTTAAGGGTTACTTG	250	NM_010548.2
	R: AGACACCTTGGTCTTGGAGCTTA		
IL-4	F: GGAGGCATGTTCGGTAGTGG	134	NM_021283.2
	R: CCCTGCGTTGGATTTCGTG		
IL-12	F: CAGCATGTGTCAATCACGCTAC	158	MMU23922
	R: TGTGGTCTTCAGCAGGTTTC		
TGF-β	F: CTGCTGACCCCCACTGATAC	168	NM_001001606.1
	R: GGGGCTGATCCCGTTGATT		
COX2	F: CTCACGAAGGAACTCAGCAC	150	NC_012387.1
	R: GGATTGGAACAGCAAGGATTTG		
MUC2	F: T CTGACCAAGAGCGAACACAA	310	NM_023566.4
	R: CATGACTGGAAGCAACTGGA		
ZO-1	F: GCGAACAGAAGGAGCGAGAAGAG	112	NM_001163574.2
	R: GCTTTGCGGGCTGACTGGAG		
Claudin-1	F: TGTCCACCATTGGCATGAA	163	NM_016674.4
	R: CTAATGTCGCCAGACCTGAAA		
LRP5	F: GCCATTGAAAACCCTGCTGA	211	NM_008513.3
	R: AGCGCAATGGTAGGTAGGTT		
PCNA	F: GAAGTTTTCTGCAAGTGGAGAG	157	NM_011045.2
	R: CAGGCTCATTCATCTCTATGGT		
Occludin	F: AGCAGCAATGGTAACCTAGAG	143	NM_001360536.1
	R: CACCTGTCGTGTAGTCTGTTTC		
GAPDH	F: AGGAGAGTGTTTCCTCGTCC	187	NM_204305.2
	R: TGCCGTGAGTGGAGTCATAC		

### Cell culture

The IEC-6 cell line was maintained in DMEM supplemented with 10% fetal bovine serum (FBS) and 1% penicillin–streptomycin at 37 °C in a 5% CO_2_ atmosphere. Cells were passaged upon reaching approximately 90% confluency. The culture medium was aspirated, and the cells were washed with pre-warmed, sterile PBS. Adherent cells were detached using 1 mL of 0.25% trypsin at 37 °C for 2 min, with digestion monitored microscopically until the cells appeared small, spherical, and refractile. Digestion was terminated by adding 2 mL of complete medium, followed by centrifugation at 1000 × g for 5 min. The pellet was resuspended in 3 mL of complete medium and transferred to new culture vessels.

According to the experimental scheme, drugs were prepared in the culture medium in advance, in which the CORT concentration gradient was 200, 400, 600, 800, and 1000 μM, and the NaBu concentration gradient was 0.25, 0.5, 1, 2.5, and 5 mM, and they were filtered and sterilized by a 0.22 μm sterile filter. When the cells grow to 80% fusion, the complete medium containing different concentrations of CORT and NaBu is added according to the experimental requirements, and then the culture is continued. Follow-up experiments were carried out after cell treatment.

### CCK-8

The effect of CORT and NaBu on cell viability and proliferation was evaluated using the CCK-8 assay. Cells were divided into three groups: (1) a blank control group containing complete medium and CCK-8 solution without cells; (2) a negative control group consisting of cells cultured in complete medium with CCK-8 solution; and (3) an experimental group comprising cells treated with varying concentrations of CORT or NaBu in complete medium supplemented with CCK-8 solution. Both the control group and the different concentrations of CORT or NaBu treatment groups included six replicate wells. IEC-6 cells were seeded at a density of 1 × 10^4^ cells per well in 96-well plates and cultured for 24 h. Different concentrations of CORT (200, 400, 600, 800, 1000 μM) or NaBu (0.25, 0.5, 1, 2.5, 5 mM) were added at different time points (12, 24, 48, 72 h) to determine optimal drug concentration and incubation duration. After treatment, the cells were washed once with PBS, and 100 μL of serum-free medium containing 10% CCK-8 solution was added to each well. The plates were incubated at 37 °C for 40 min, and absorbance was measured at 450 nm using a microplate reader.

### Cell scratch test

IEC-6 cells were seeded in 6-well plates at a density of 4 × 10^5^ cells per well in 2 mL of complete medium and cultured at 37 °C with 5% CO_2_ for 24 h. When the cell proliferation reached more than 90% fusion, five evenly spaced horizontal lines were marked on the back of each plate. A vertical scratch was made on the cell monolayer using a 20 μL pipette tip, ensuring perpendicularity to the horizontal lines. The intersections of the horizontal lines and the scratch were served as observation points for analysis. After washing with PBS, the control group was treated with 2 mL of low-serum medium, while the CORT group was treated with 2 mL of low-serum medium containing 400 μM CORT, and the CORT + NaBu group was treated with 2 mL of low-serum medium containing 400 μM CORT and 1 mM NaBu. Initial images (0 h) were captured immediately under a microscope, with subsequent observations recorded every 12 h. Scratch width changes were measured using ImageJ software, and cell migration rates were calculated.

$$\mathrm{Cell}\;\mathrm{mobility}\;\left(\%\right)\;=\;\frac{0\;\mathrm h\;\mathrm{Scratch}\;\mathrm{area}-\mathrm{Final}\;\mathrm{scratch}\;\mathrm{area}\;}{0\;\mathrm h\;\mathrm{Scratch}\;\mathrm{area}}\;\times\;100\%$$

### Flow cytometry

Following the experimental protocol, cells were treated with the designated drugs. Adherent cells were detached using trypsin without EDTA and collected. The cell suspension was centrifuged at 4 °C for 5 min at 1800 × g to pellet 1 × 10^5^ to 5 × 10^5^ cells. To prevent false positives, digestion time was carefully controlled. Cells were washed twice with pre-cooled PBS, with each wash followed by centrifugation at 4 °C for 5 min at 1800 × g. The cell pellet was resuspended in 100 μL of 1 × Binding Buffer and gently agitated to ensure complete suspension. Subsequently, 5 μL of Annexin V-FITC and 5 μL of PI staining solution were added, and the mixture was incubated at room temperature (20–25 °C) in the dark for 10 min. After adding 400 μL of 1 × Binding Buffer, the samples were gently mixed and analyzed by flow cytometry within one hour.

### 16S rDNA sequencing

The genomic DNA was extracted from cecum contents using the CATB method, with the purity and concentration subsequently assessed via agarose gel electrophoresis. An aliquot of the extracted DNA was then transferred to a centrifuge tube and diluted to a concentration of 1 ng/μL using sterile water. The V3-V4 hypervariable region of the 16S rRNA gene was targeted for amplification using the universal primer pair 341F (5′-CCTACGGGRSGCAGCAG-3′) and 806R (5′-GGACTACVGGGTATCTAATC-3′). PCR amplification was performed using Phusion® High-Fidelity PCR Master Mix with GC Buffer (New England Biolabs, Ipswich, MA, USA), which incorporates high-efficiency and high-fidelity enzymes to ensure optimal amplification efficiency and accuracy. Following amplification, the Next® UltraTM DNA Library Prep Kit for Illumina (New England Biolabs) was employed for library construction. The resulting libraries were quantified using the Qubit system and subjected to quality control. Libraries meeting the quality standards were subsequently sequenced on the Illumina MiSeq platform (Novogene, Beijing). The 16S rRNA sequencing data were processed and analyzed using the Beijing Novogene cloud platform. Significantly changed floras were identified based on the following criteria: variable importance in projection (VIP) > 1.0, fold change (FC) > 1.2 or FC < 0.833, and statistical significance (*p* < 0.05).

### Statistical analysis

The results are presented as mean ± SEM based on at least three independent experiments. The statistical software of SPSS 26.0 was used to perform the analysis of the data collected. The Student’s t-test (parametric test, unpaired test) was used to determine significance between two groups. One-way ANOVA followed by Bonferroni correction and Duncan post hoc test were performed for multiple comparisons. Normality of data distribution was verified using the Shapiro–Wilk test. All data met the assumptions of ANOVA and t-tests. *P* < 0.05 was considered statistically significant, ^*^P < 0.05, ^**^P < 0.01 and ^***^P < 0.001 between CON and CORT groups, and ^#^*P* < 0.05, ^##^*P* < 0.01 and ^###^*P* < 0.001 between CORT and CORT + NaBu groups.

## Data Availability

The datasets analyzed during the current study are available from the corresponding authors upon reasonable request.

## Abbreviations

ADFI: Average daily feed intake
ADG: Average daily gain
AKP: Alkaline phosphatase
ALB: Albumin
ALT: Alanine aminotransferase
AST: Aspartate aminotransferase
BSA: Bovine serum albumin
BWG: Body weight gain
CCIS: Chronic corticosterone-induced stress
CD: Crypt depth
CORT: Corticosterone
DDH_2_O: Distilled deionized water
DMSO: Dimethyl sulfoxide
FBS: Fetal bovine serum
F/G: Feed conversion rate
GLB: Globulin
GLU: Glucose
HDACi: Histone deacetylase inhibitors
HDL-C: High-density lipoprotein cholesterol
H&E: Hematoxylin and eosin
HPA: Hypothalamic–pituitary–adrenal
IECs: Intestinal epithelial cells
LDL-C: Low-density lipoprotein cholesterol
LEfSe: Linear discriminant analysis effect size
NaBu: Sodium butyrate
NMDS: Non-metric multidimensional scaling
OTUs: Operational taxonomic units
PAS: Periodic acid-Schiff
PBS: Phosphate-buffered saline
PCA: Principal component analysis
PCoA: Principal coordinates analysis
RT-qPCR: Reverse transcription-quantitative polymerase chain reaction
SCFAs: Short-chain fatty acids
TC: Total cholesterol
TG: Triglycerides
TP: Total protein
VCR: The ratio of VH to CD
VH: Villus height
VSA: Villus surface area
ZO-1: Zonula occludens-1
